# Effects of opium on cholesterol metabolism in rats fed normal and high-fat/high-cholesterol diet

**DOI:** 10.1016/j.toxrep.2025.102014

**Published:** 2025-03-26

**Authors:** Elaheh Ebrahimi, Iraj Khodadadi, Gholamreza Shafiee, Ebrahim Abbasi

**Affiliations:** a Department of Clinical Biochemistry, School of Medicine, Hamadan University of Medical Sciences, Hamadan, Iran; b Nutrition Health Research Center, Institute of Health Sciences and Technologies, Hamadan University of Medical Sciences, Hamadan, Iran

**Keywords:** Opium, Lipid, Cholesterol, Rat

## Abstract

There is a misconception that opium can lower blood sugar and cholesterol levels. Hence, this study aimed to investigate the influences of opium on the expression of key cholesterol metabolism genes in the liver and intestine of rats receiving a cholesterol-rich diet. Male Wistar rats were randomly divided into four groups (n = 6): normal control, opium addiction, hypercholesterolemic diet, and opium addiction received hypercholesterolemic diet. After 28 days, the blood glucose levels, liver enzymes, and cholesterol in the rat's serum were measured. The cholesterol regulatory genes and transporters such as 3-hydroxy-3-methylglutaryl coenzyme A (HMG-CoA) reductase, low-density lipoprotein receptor (LDL-R), cholesterol 7 alpha-hydroxylase 1 (CYP7A1) (in liver tissue), and ATP Binding cassette subfamily g member 5 and 8 (ABCG5 and ABCG8), and Niemann-Pick C1-like 1 protein (NPC1L1) (in intestinal tissue) were measured. Intestinal morphological changes were also evaluated. Opium decreased serum glucose and total cholesterol levels (P < 0.05). In contrast, the levels of liver enzymes increased compared to the normal control group (P < 0.05). Histological examinations revealed that opium caused disorganization, deformation, and destruction of cells in intestinal tissue. Real-time PCR analysis demonstrated that opium increased the expression of LDL receptor genes, HMG-CoA reductase enzyme, and CYP7A1 in the liver compared to the normal control group (P < 0.05). The changes of ABCG8 and NPC1L1 transporters in intestinal tissue were not significant. Opium had beneficial effects on blood lipid and glucose levels, but histological findings indicated destructive effects on intestinal tissues.

## Highlights

• High fat and high cholesterol diets had deteriorated effect of liver function in addicted animals.
• High fat and high cholesterol diets had deteriorated effects on intestinal structure in addicted animals.
• Opium increases the low-density lipoprotein receptor (LDL-R) gene expression.
• Opium increases 3-hydroxy-3-methylglutaryl coenzyme A (HMG-CoA) reductase gene expression.
• Opium increases cholesterol 7 alpha-hydroxylase 1 (CYP7A1) reductase gene expression.

## 1 Introduction

Drug abuse is one of the most frequent threads to humans which can lead to disability and even early death [1]. Addiction is known to be a prevalent problem in many countries, especially in the Middle East. Usage of synthetic additives is common in Western countries but traditionally, opioid is commonly used in Iran [2]. Since ancient times, *Papaver somniferum L*, which is known as opium poppy has been cultivated for opium (the milky latex) and poppy seeds [3]. Many people believe that opium is effective in the treatment or prevention of cardiovascular disorders (CVD). Hence, studies have shown that opium usage in patients suffering from chronic illnesses such as hypertension, diabetes, and ischemic heart disease is more than in the general population [4], [5]. Clinical observations have shown that opium can elevate the atherosclerotic plaque formation’s risk and negatively impact the lipid profile of the animal models [2].

Dietary cholesterol is found in cheese, shrimp, pork, beef, egg yolks, butter, poultry, and increases LDL-C. Dietary cholesterol can decrease hepatic LDL-R, declining the clearance of blood cholesterol [6]. Cholesterol is a lipophilic molecule that is vital for human life. Due to the lipophilic nature of cholesterol, it is transported within lipoprotein particles such as high-density lipoprotein cholesterol (HDL-C), low-density lipoprotein cholesterol (LDL-C), and triglycerides. However, a high fat/cholesterol diet can induce different tissue injuries and increase the risk of CVD, cognitive deficit, and non-alcoholic fatty liver disease (NAFLD) [7], [8].

Diets in which more than 30 % of energy intake is from fat induce obesity [9]. Mami et al. showed that oral consumption of opium for 60 days increased fasting blood sugar. Their research proved that opium caused an elevation of total cholesterol, triglycerides, and LDL-C compared with healthy rabbits [10]. Sanli et al., showed triglycerides were higher in ethyl glucuronide groups than normal subjects, meanwhile, HDL-C, LDL-C, and cholesterol levels were not significantly different [2]. Salman et al., showed higher LDL-C, Cholesterol, and triglyceride in female and male opium and bhang users compared to controls [11].

The enzyme 3-hydroxy-3-methylglutaryl co-reductase (HMG-CoA reductase) is the rate-limiting enzyme of the cholesterol biosynthesis pathway and plays the main role in controlling cholesterol synthesis [12]. Recent studies have shown that the Niemann-Pick C1-like 1 protein (NPC1L1) protein is located on the surface of enterocytes and is important for absorbing cholesterol in the intestines. ATP-binding cassette (ABC) transporters G member 5 and 8 (ABCG5 and ABCG8) help to remove plant sterols and cholesterol from enterocytes and expel them back into the intestine, which explains why some types of sterols are poorly absorbed. These findings indicate that cholesterol absorption is a complex process controlled by multiple enterocyte genes. The efficiency of cholesterol absorption depends on the balance between the intake and removal of cholesterol in the intestines [13].

Considering that opium increases blood cholesterol and LDL-C in the hypercholesterolemic model, it may affect the main pathways of cholesterol, including liver synthesis, liver harvest, or change the conversion of cholesterol into bile acids, so this study aimed to investigate the effect of opium on the expression of HMG-CoA reductase, cholesterol 7-alpha-hydroxylase (CYP7A1), LDL receptor (LDL-R) in the liver and NPC1L1, ABCG5 and ABCG8 genes in the intestine of addicted rats

## 2 Methods

### 2.1 Animals

In this study, 24 male Wistar rats (200–220 g; 8 weeks old) were obtained from Hamadan University of Medical Sciences (Hamadan, Iran). Rats had free access to water and standard food (chow diet). The rats were acclimatized to animal house conditions for 10 days and then were divided randomly into 4 groups (n = 6 rats in 3 laboratory animal cages), including; group 1; normal control (NC)(received normal chow diet), group 2: hypercholesterolemic or high cholesterol diet (HC, received 1 % cholesterol along with 0.5 % cholic acid and 20 % corn oil), group 3: normal rats received opium (OP) (received 40 mg/kg opium twice daily via gavage for 4 weeks, and group 4: opium (OP) (received 40 mg/kg opium twice daily via gavage for 4 weeks) + hypercholesterolemic (HC)(received 1 % cholesterol along with 0.5 % cholic acid and 20 % corn oil). Opium-addicted animals received opium (40 mg/kg) twice daily via gavage for the entire 4 weeks based on previously published papers [14].

### 2.2 Sample preparation

Following a 4-week study period, after overnight fasting the rats were weighed, and anesthetized by intraperitoneal (IP) injection of xylazine (10 mg/kg) and ketamine (75 mg/kg), and blood was collected from the inferior vena cava. The blood was collected and centrifuged at 5000*g* for 10 min to prepare the serum. The serum was used for the assessment of liver enzymes and total cholesterol. The liver and intestine tissues were removed, weighed, and stored at −80 °C for gene expression.

### 2.3 Biochemical factors

Total cholesterol (TC), fasting blood sugar (FBS), alanine aminotransferase (ALT), and aspartate aminotransferase (AST), in serum were measured using the enzymatic method (Pars Azmoon, Iran) with an AutoAnalyzer.

### 2.4 Real time-PCR

The total RNA was extracted using kit (Kiazool Co., Iran). To check the quantity and contamination with phenol and protein, the absorbance of RNA was measured at wavelengths of 230, 260, and 280 nm using NanoDrop. The ratios of 260/280, and 260/230 were acceptable and indicated the appropriate quantity and purity of the sample. Also, 1 % agarose was used to detect the quality of RNA. cDNA synthesis was done by using the cDNA Synthesis Kit (Parstos Biotechnology, Iran). The annealing temperature of primers was determined and then cDNA was used as a template for the Real-time PCR reactions. The SYBER premix Ex Taq (Takara Bio) was used for PCR reactions. This kit uses SYBR green as a fluorescent dye. SYBR green is a dye that binds to double-stranded DNA (dsDNA) and creates fluorescence. Hence, when DNA is amplified, it emits fluorescence which is recorded by the real-time detector (Roche, LightCycler® 96). The recorded fluorescence intensity shows the concentration of dsDNA. To do the PCR reaction, 5 µl of SYBR green, 1 µl of cDNA, 0.5 µl of forward primer, 0.5 µl of reverse primer, and 3 µl of nuclease-free water were used (total volume 10 µl). Glyceraldehyde 3 phosphate dehydrogenase (GAPDH) was used as a housekeeping gene. The PCR reactions were done as follows: preincubation at 95 °C for 300 s, denaturation at 95 °C for 15 s, annealing for GAPDH at 62 °C, NPC1L1 at 62 °C, ABCG5 at 62 °C, ABCG8 at 62 °C, LDL-R at 60 °C, HMG-CoA reductase at 60 °C, CYP7A1 at 60 °C for 30 s (30 s for all genes), extension at 72°C for 30 s, and melting at 72°C for 10 s, 65°C for 60 s, and 97°C for 1 s. [15], [16]. Table 1 shows the primer sequence.

**Table 1:** The primer sequences used in this study.

**Gene**	**Sequence**	
GAPDH	F	AAGGTCGGTGTGAACGGATTTGG
	R	TCCTGGAAGATGGTGATGGGTT
NPC1L1	F	TCCTCCTGCTGTCGCCTTTAT
	R	TACGCTGCTAGACCCCCTTTG
ABCG5	F	AACGCTGTGAACCTCTTTCCC
	R	CCAGTAACACACGCTGCTGAA
ABCG8	F	TTTACCACCCTGATCCGTCGT
	R	GTGGCCGTAGTAAAGGAAGCC
LDL-R	F	AAGCCATTTTCAGTGCCAACC
	R	CTCACACCAGTTTACCCCTCT
HMG-CoA R	F	TCTTCAGCACTGTCGTCATTC
	R	CCCTTACTTCATCCTGTGAGTTT
CYP7A1	F	GAGGGATTGAAGCACAAGAACC
	R	ATGCCCAGAGAATAGCGAGGT

GAPDH: Glyceraldehyde 3 phosphate dehydrogenase, LDL-R: low-density lipoprotein receptor, HMG-CoA R: 3-hydroxy-3-methylglutaryl coenzyme A reductase, CYP7A1: cholesterol 7 alpha-hydroxylase 1, NPC1L1: Niemann-Pick C1-like 1 protein, ABCG5: ATP binding cassette subfamily g member 5, ABCG8: ATP Binding cassette subfamily g member 8.

### 2.5 Histological examination

For histological examination, the intestine of each rat was removed and rinsed with phosphate buffer saline (PBS) to remove any debris. Then the tissue was fixed in 10 % formalin at room temperature. After 24 h, the formalin was replaced, and the sample was transferred to the histology laboratory for tissue section preparation. Fixed intestinal tissue was embedded in paraffin during various steps, including dehydration by graded ethanol series (60 %, 70 %, 90 %, and 100 % ethanol) for one hour. The sample was cleared by xylene to remove ethanol. The section at a thickness of 5 µm was prepared from the embedded sample using a microtome and mounted on glass slides. The sample was deparaffinized in xylene for 10 min and rehydrated in water for 5 min. The slide was stained with hematoxylin solution for 10–20 min and then rinsed in tap water. The slide was rinsed briefly in 10 % acetic acid for a few seconds and then in ethanol (85 %) for one minute. Then, the sample was stained with eosin for 5–15 min and rinsed in water. The slide was dehydrated using an ascending ethanol concentration (60 %, 70 %, 90 %, and 100 % ethanol) for 5 min each and then cleared by xylene for a few minutes. The sample was mounted and then covered with a glass coverslip. The stained section was examined under a light microscope (Nikon E800, Japan) for histopathological evaluation. About 20 non-overlapping fields were randomly examined from each group by light microscopy [17]. The severity of the intestinal injury was determined by using a histological grading system according to the previously published paper [18].

### 2.6 Statistical analysis

Data analysis was done using SPSS software (version 26). The data were presented as Mean ± SEM. To assess normal distribution, the Shapiro-Wilk test was employed. Once normal distribution and homogeneity of the groups were confirmed, a one-way ANOVA followed by Tukey's test was conducted to compare the groups. Statistical significance was determined at P < 0.05. Graphs were prepared using GraphPad Prism software (version 9).

## 3 Results

### 3.1 Body weight

**Fig. 1:**
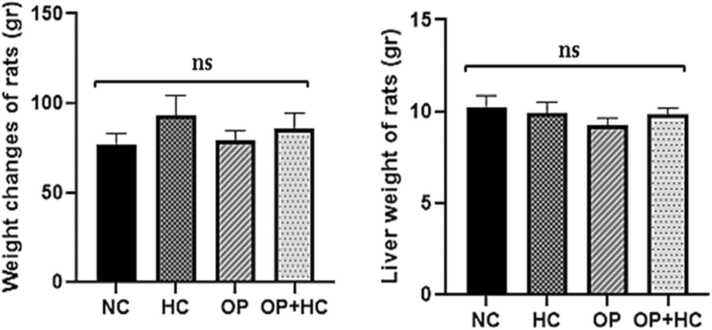
Changes in the weight of rats and their liver weight in the studied groups. The results are reported as Mean ± SEM. ns: non-significance compared to the normal control group and comparison between groups. NC: normal control, HC: high cholesterol, OP: opium-addicted, and OP + HC: opium-addicted + high cholesterol, gr: gram.

The weight of the rats elevated in the hypercholesterolemic (HC) group compared to the normal control group (NC), although this increase was not statistically significant. Furthermore, the change in liver weight was not significant among the treated groups. However, the opium (OP) group exhibited a decrease in liver weight than the other groups. Nonetheless, this decrease was not statistically significant (Fig. 1).

### 3.2 Biochemical factors

FBS levels for each group are displayed in Fig. 1. Serum FBS concentrations were significantly lower in the opium group (OP) and the opium + hypercholesterolemic group (OP+HC) than in the normal control group (NC) (P < 0.01 and P < 0.05, respectively). As compared to the OP+HC group, this reduction was more in the OP group. FBS reduction was not statistically significant in the hypercholesterolemic (HC) group when compared to the normal control group (NC)(P < 0.05).

**Fig. 2:**
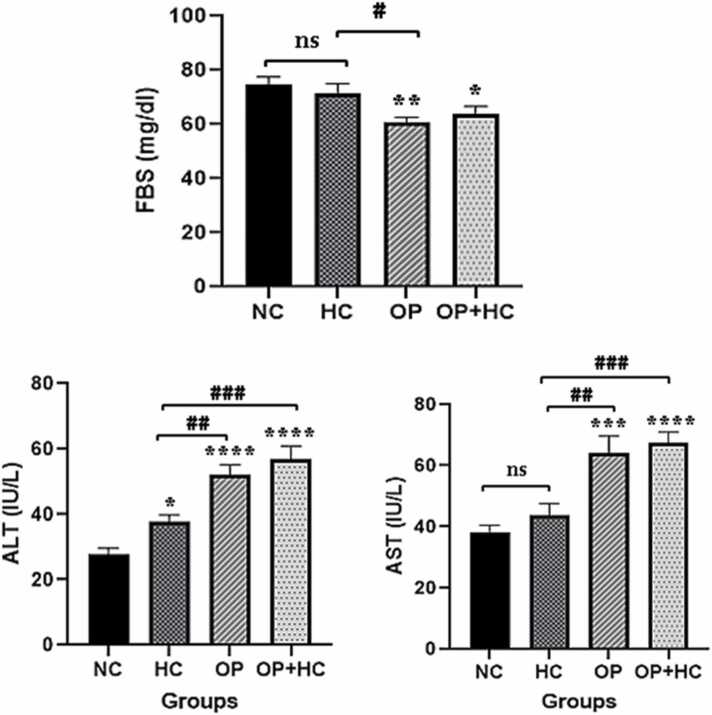
Serum levels of fasting blood sugar and liver enzymes in the studied groups. The results are shown as Mean ± SEM. ns: no significance compared to the control group. *: p < 0.05, **: p < 0.01, ***: p < 0.001, and ****: p < 0.0001 compared to the normal control group. #: p < 0.05, ##: p < 0.01, and ###: p < 0.001 compared to specific group. NC: normal control, HC: high cholesterol, OP: opium-addicted, and OP + HC: opium-addicted + high cholesterol, FBS: fasting blood sugar, AST: aspartate aminotransferase, ALT: alanine aminotransferase.

The levels of ALT increased in all study groups compared to the NC group (P < 0.05). The ALT activity increased in the OP and OP + HC groups compared to the control (P < 0.01 and P < 0.01, respectively). The ALT activity increased in the OP and OP + HC groups compared to the HC group (P < 0.01 and P < 0.001, respectively). The activity of AST also increased in the OP and OP + HC groups compared to the NC group (P < 0.001 and P < 0.0001, respectively)(Fig. 2).

The total cholesterol in the serum increased significantly in the HC group (170.33 ± 17.10) compared to the NC group (99.33 + 10.9) (P < 0.001). Opium-treated (OP) animals (70.2 ± 9.10) showed reduced total cholesterol levels compared to the NC group (P < 0.05). Furthermore, the OP + HC group showed a reduced total cholesterol level (123.6 ± 5.35) compared to the HC group (P < 0.001).

### 3.3 Gene expression

Changes in the expression patterns of the cholesterol regulatory genes were assessed using the 2^-∆∆Ct^ method. The expressions of LDL-R, HMG-CoA-Reductase, and CYP7A1 genes in liver tissue and the expression level of ABCG5, ABCG8, and NPC1L1 genes in intestinal tissue were evaluated.

**Fig. 3:**
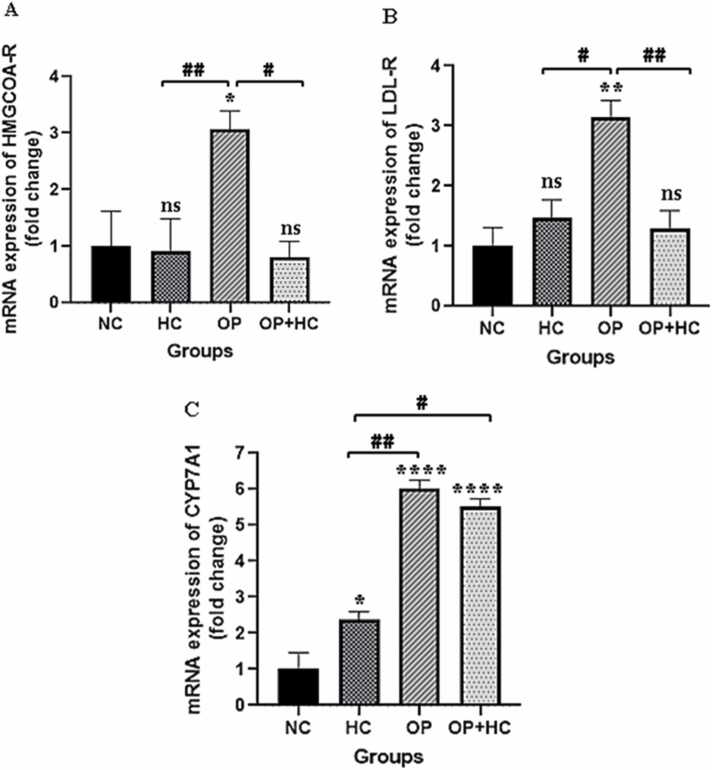
The results of examining LDL-R, HMG-CoA-R, and CYP7A1 gene expression in the liver of the studied groups. The results are reported as Mean ± SEM. ns: no significance compared to the control group, *: p < 0.05, **: p < 0.01, ****: p < 0.0001 compared to the normal control group, #: p < 0.05, ##: p < 0.01 compared to specific group. NC: normal control, HC: high cholesterol, OP: opium-addicted, and OP + HC: opium-addicted + high cholesterol, LDL-R: low-density lipoprotein receptor, HMG-CoA R: 3-hydroxy-3-methylglutaryl coenzyme A reductase, CYP7A1: cholesterol 7 alpha-hydroxylase 1.

Based on the real-time-PCR results in Fig. 3, the OP group had significantly higher HMG-CoA reductase gene expression than NC group (P < 0.05). There was a three-fold increase in gene expression as compared to the normal control group.

A notable rise in LDL-R gene expression was observed in the OP group in comparison to the NC group (P < 0.01). Statistical analysis revealed that this increase in gene expression was one-third of that seen in the NC group. No significant difference was found in the gene expression of this enzyme in the OP + HC group when compared to the NC group (Fig. 3).

The gene expression level of the CYP7A1 enzyme among the studied groups exhibited a significant increase (P < 0.05) when compared to the NC group. The increase in CYP7A1 gene expression was 6 times in the OP group, 2.3 times in the HC group, and 5.5 times in the OP+HC group in comparison to the control group (Fig. 3).

The expression level of the NPC1L1 transporter gene did not show any significant differences among the studied groups (HC, OP, and OP+HC) when compared to the NC group. The expression level of the ABCG5 transporter gene in the HC group displayed a significant increase (P < 0.05) compared to the NC group, while its expression was reduced in opium OP and OP+HC groups compared to the hypercholesterolemic group (P < 0.01 and P < 0.05, respectively).

The statistical analysis revealed no significant change in the ABCG8 transporter gene levels in treated groups when compared to the NC group. A non-significant rise in the expression of the ABCG8 transporter gene was observed in the opium addict group (OP) in comparison to the NC group.

### 3.4 Histopathological examination

**Fig. 4:**
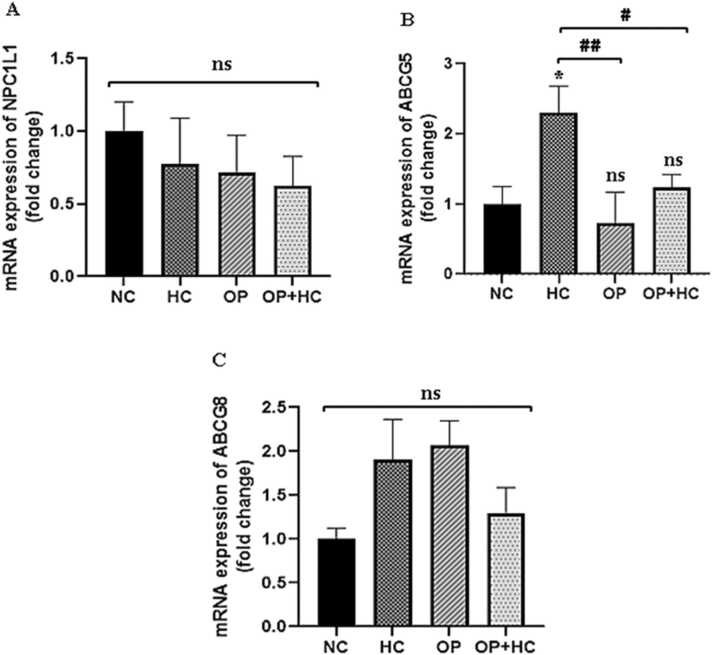
The results of examining NPC1L1, ABCG5, and ABCG8 gene expression in the intestines of the studied groups. The results are reported as Mean ± SEM. ns: no significance compared to the control group, *: p < 0.05 compared to the normal control group, #: p < 0.05, ##: p < 0.01 compared to compared to specific group. NC: normal control, HC: high cholesterol, OP: opium-addicted, and OP + HC: opium-addicted + high cholesterol, ABCG5: ATP binding cassette subfamily g member 5, ABCG8: ATP Binding cassette subfamily g member 8, NPC1L1: Niemann-Pick C1-like 1 protein.

**Fig. 5:**
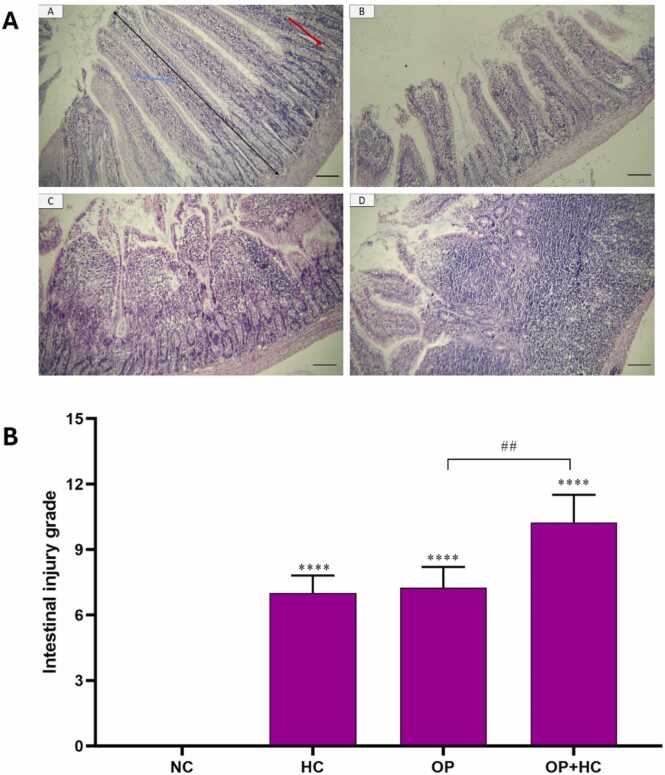
Hematoxylin-eosin (H&E) staining of different treated groups (A) and the severity of the intestinal injury (B) in different groups. A: Histological changes in the intestine in control groups (A), group receiving hypercholesterol diet (B), group receiving opium (C), group receiving opium and hypercholesterol diet (D)(10x magnification, scale bar: 20 µm). Blue arrow: villi, red arrow: Crypts of Lieberkuhn, and black arrow: villi length. B: The severity of the intestinal injury. ****: p < 0.0001 compared to the normal control group, #: p < 0.05, ##: p < 0.01 compared to compared to specific group. NC: normal control, HC: high cholesterol, OP: opium-addicted, and OP + HC: opium-addicted + high cholesterol.

The histopathological examination of the intestine in the studied groups is depicted in Fig. 5. In the NC, the slides revealed a normal tissue structure characterized by finger-shaped villi and normal epithelial cells with a striated border. The crypts of Lieberkuhn were also observed to be normal. On the other hand, the administration of a high-cholesterol diet (HC) resulted in a significant reduction in villi length. In the group receiving opium, not only was there a substantial decrease in villi length, but the shape of the villi also became irregular. Additionally, the ratio of absorptive cells to mucous glands decreased, and there was a notable infiltration of lymph cells into the axis of the villi. The severity of tissue damage was even more pronounced in both OP and HC groups. In this group, apart from the destruction of the normal villi structure, the striated border of absorptive cells was greatly reduced, and there was a high intensity of lymph cell infiltration into the axis of the villi.(Fig. 4).

## 4 Discussion

The findings of this investigation showed that the use of opium leads to changes in the structure of the intestines at a microscopic level. Furthermore, an analysis of various biochemical factors indicated that the OP group exhibited a decrease in total cholesterol and fasting blood sugar levels compared to the HC group. Moreover, the levels of ALT and AST enzymes were elevated in all groups when compared to the NC group. The weight of the rats increased in all groups following 28-day exposure to opium and a hypercholesterolemic diet when compared to the normal control group, but this increase was not statistically significant. As a result, it appears that neither the hypercholesterol diet nor opium addiction affects appetite. The analysis of the rats' liver weight revealed that opium non-significantly reduced liver weight.

An examination of gene expression patterns demonstrated that opium usage upregulates the expression of the HMG-CoA reductase and LDL receptor genes in the liver of the opium (OP) treated group. Additionally, the expression of the CYP7A1 gene was enhanced in the OP and OP+HC groups. Moreover, opium was found that only the expression of the ABCG5 gene was altered in the OP and OP+HC groups when compared to the HC group.

Sadeghian et al. demonstrated that in rats with induced diabetes, the use of opioids had no discernible impact on blood lipid profile or glucose metabolism [19]. According to a prior study, opium may lower blood sugar, but there was no change in HbA1C, triglyceride, total cholesterol, LDL-C, and HDL-C levels [20]. Mohammadi et al., findings showed that opium usage can have worsening impacts on atherosclerosis plaque formation, which is related to hypercholesterolemia, mostly impacting lipid profile [21]. According to a Hosseini et al., study, opium had no effect on FBS, total cholesterol, LDL-C, or HDL-C between the two groups it was investigated in, and it solely lowers TG in addicts compared to non-addicts [22]. The Gozashti et al. investigation revealed that opium does not appear to have a significant effect on blood glucose and insulin resistance [23]. The findings of this study indicate that rats exposed to opium showed a significant reduction in total cholesterol and FBS compared to the hypercholesterolemic rats. Hence, some studies reported that opium reduced blood glucose and cholesterol levels, while others reported that opium reduced these factors[24]. These conflicting results may be due to the different animal models, variations in sample size, type of opium used, method of consumption (oral, inhalation, etc.), duration of opium exposure and purity, and dosage of opium. Animals have shown that morphine affects glucose. Numerous mechanisms have been proposed to explain its effects, including an increase in the counter-regulatory hormones glucagon, cortisol, adrenocorticotrophic hormone (ACTH), noradrenalin, and plasma adrenalin. These hormones raise blood glucose levels [25]. Other studies showed that opium users had reduced lipid levels; however, this could have been caused by weight loss or an unhealthy diet rather than an actual effect of opium. Individual characteristics include underlying medical conditions, psychological issues, degree of physical exercise, and participant age range, etc [26].

We evaluated the expression of important genes related to cholesterol metabolism. The first crucial component is the enzyme HMG-CoA reductase, which is the limiting enzyme of the mevalonate metabolism pathway and is the limiting enzyme for cholesterol synthesis [27]. Statins, or competitive inhibitors of HMG-CoA reductase, are the main medication used to treat hypercholesterolemia [28]. Our results demonstrate that the gene expression of this enzyme increased three times in the opium addict group alone when compared to the normal control group; no significant changes were observed in the other groups. As a result, opium by itself can boost this enzyme's gene expression and hence has an impact on cholesterol production. In the current investigation, we showed that the LDL receptor gene expression level was only three times higher in the opium addict group as compared to the normal control group. In this study, by examining the gene of this enzyme in the studied groups, it was concluded that in all groups, the expression of the CYP7A1 enzyme gene was increased compared to the normal control group, and this increase was found in the opium-addicted and cholesterol-rich diet groups and opium addicts were more than the group receiving cholesterol-rich diet. This study looked at these genes in the liver and the expression of the ABCG5, ABCG8, and NPC1L1 genes in the rats' jejunum. Opium has not been able to alter the expression of ABCG8, and NPC1L1. The ABCG5 gene expression was reduced in opium and opium hypercholesterolemic groups compared to the hypercholesterolemic group.

Despite extensive opioid use, its effect on cholesterol metabolism has not been extensively studied. Hence, the exact mechanisms are not determined. Previous studies reported that addiction may induce hypolipidemia through lifestyle factors, such as reduced appetite, nutritional deficiency, malnutrition, and weight loss [29].

Our results showed that opium increased the CYP7A1 and LDL receptors. Increasing the expression of CYP7A1 leads to cholesterol excretion through bile acids, that decrease hepatic cholesterol levels. As mentioned above, LDL receptors uptake cholesterol from the blood into the liver, which can reduce blood cholesterol levels. Interestingly, when opium reduces blood cholesterol, the liver may try to compensate and increase HMG-CoA reductase to produce cholesterol levels. In the intestine, opium also reduces the ABCG5, which can lead to increased cholesterol absorption. ABCG5/ABCG8 transporters pump sterols (plant sterols and cholesterol) out of intestinal cells back into the intestinal lumen. Hence, decreased ABCG5 levels can decrease the excretion of sterols into the intestinal lumen. Although the levels of ABCG5 were reduced in addicted animals, the changes of ABCG8 and NPC1L1 were not significant compared to hypercholesterolemic or control groups. The results of this meta-analysis conducted by Ojo et al., showed that opium significantly reduced cholesterol levels in diabetic patients [30].

Histological analysis of this study revealed that the opium-treated groups showed a significant change in intestinal structure. The group given opium and a hypercholesterolemic diet had significantly more severe intestinal damage. The relationship between intestinal injury and addiction has been studied formerly. Shavakhy et al., have established a correlation between opium consumption and liver damage, particularly among individuals diagnosed with hepatitis C. Previous research has extensively documented the detrimental impact of addiction to this drug on the structure and function of various body systems [31]. Monfared et al., showed that opium has detrimental effects on the liver and kidney tissues of rabbits. Furthermore, prolonged exposure to opium for two consecutive months in New Zealand white rabbits leads to multiple structural modifications in the liver tissue. Additionally, the hyperemic liver tissue near the central lobular vein displays leukocyte cell infiltration, fat droplet accumulation, hepatocyte necrosis, and liver sinusoid dilation [32]. As a result, the most noticeable alterations in this group were the irregularities in the hepatocyte arrangement and the changes in the size and shape of the central vein. In this experiment, opium induces intestinal injury probably via various pathways, including: 1) opium was administrated orally, so it probably directly affects intestine structure. 2) opium can increase intestinal permeability, lead to villous atrophy, and induce epithelial cell apoptosis. 3) opium can induce oxidative stress in the intestine. 4) opium can induce intestinal inflammation and leukocyte infiltration. 5) opium can induce gut dysbiosis (imbalance of gut microorganisms) [33], [34], [35], [36], [37].

This study had some limitations. Opium showed cholesterol-lowering effects. However, we did not use lipid-lowering agents to compare the hypocholesterolemic effect with opium. Since opium consists of many alkaloids, it is essential to determine the useful and hypocholesterolemic constituents. Hence, we cannot judge what component is useful. Furthermore, the lack of adjustment for cigarette smoking was another limitation of this experiment.

## 5 Conclusion

In this experiment, opium decreased serum total cholesterol and glucose levels. It increased the expression of LDL receptor, HMG-CoA reductase enzyme, and CYP7A1 in the liver compared to the hypercholesterolemic group. Histological findings indicated that opium has harmful effects on intestinal tissue.

## Ethical approval

The Animal Care Committee at Hamadan University of Medical Sciences (Hamadan, Iran, Ethics code: IR.UMSHA.REC.1400.853) approved all protocols of this study.

## Funding

This study was funded by the 10.13039/501100004697Hamadan University of Medical Sciences (No: 1401011681).

## CRediT authorship contribution statement

**Ebrahim Abbasi:** Writing – review & editing, Visualization, Validation, Supervision, Resources, Project administration, Funding acquisition, Data curation. **Gholamreza Shafiee:** Writing – review & editing, Writing – original draft, Visualization, Validation, Supervision, Software, Investigation, Conceptualization. **Iraj Khodadadi:** Writing – original draft, Visualization, Validation, Supervision, Software. **Elaheh Ebrahimi:** Writing – original draft, Visualization, Validation, Methodology, Investigation, Funding acquisition, Formal analysis, Conceptualization.

## Declaration of Competing Interest

The authors declare the following financial interests/personal relationships which may be considered as potential competing interests: Ebrahim Abbasi reports financial support was provided by Hamadan University of Medical Sciences Faculty of Health and Research Center for Health Sciences Department of Environmental Health Engineering. Ebrahim Abbasi reports a relationship with Hamadan University of Medical Sciences Faculty of Health and Research Center for Health Sciences Department of Environmental Health Engineering that includes:. If there are other authors, they declare that they have no known competing financial interests or personal relationships that could have appeared to influence the work reported in this paper.

## Data Availability

Data will be made available on request.
